# 2,9-Bis(4-pyridylmeth­oxy)-1,10-phenanthroline

**DOI:** 10.1107/S1600536809038318

**Published:** 2009-09-26

**Authors:** Shi Guo Zhang, Long Miao Xie, Hong Li

**Affiliations:** a Department of Chemistry and Chemical Engineering, Institute of Materials Chemistry, Binzhou University, Binzhou 256603, People’s Republic of China; b Department of Chemistry, Shandong Normal University, Jinan 250014, People’s Republic of China

## Abstract

In the title mol­ecule, C_24_H_18_N_4_O_2_, the dihedral angles between the mean plane of the phenanthroline ring system and the pyridine rings are 82.52 (5) and 71.58 (4)°. The dihedral angle between the two pyridine ring planes is 53.54 (6)°. In the crystal structure, there are π–π stacking inter­actions between 1,10-phenanthroline rings, with centroid–centroid distances of 3.6101 (11) and 3.5864 (11) Å.

## Related literature

For related structures, see: Liu *et al.* (2008 ▶); Zhang & Hou (2008 ▶).
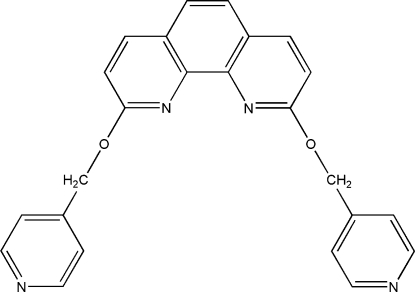


## Experimental

### 

#### Crystal data

C_24_H_18_N_4_O_2_
*M* *_r_* = 394.42
Monoclinic, 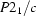

*a* = 12.701 (2) Å
*b* = 16.418 (3) Å
*c* = 9.7443 (16) Å
β = 107.535 (2)°
*V* = 1937.5 (6) Å^3^
*Z* = 4
Mo *K*α radiation
μ = 0.09 mm^−1^
*T* = 298 K
0.51 × 0.40 × 0.38 mm

#### Data collection

Bruker SMART APEX CCD diffractometer
Absorption correction: multi-scan (*SADABS*; Sheldrick, 1996 ▶) *T*_min_ = 0.956, *T*_max_ = 0.967
9860 measured reflections
3530 independent reflections
2603 reflections with *I* > 2σ(*I*)
*R*_int_ = 0.028

#### Refinement

*R*[*F* ^2^ > 2σ(*F* ^2^)] = 0.043
*wR*(*F* ^2^) = 0.113
*S* = 1.03
3530 reflections
271 parameters
H-atom parameters constrained
Δρ_max_ = 0.13 e Å^−3^
Δρ_min_ = −0.16 e Å^−3^

### 

Data collection: *SMART* (Bruker, 1997 ▶); cell refinement: *SAINT* (Bruker, 1997 ▶); data reduction: *SAINT*; program(s) used to solve structure: *SHELXTL* (Sheldrick, 2008 ▶); program(s) used to refine structure: *SHELXTL*; molecular graphics: *SHELXTL*; software used to prepare material for publication: *SHELXTL*.

## Supplementary Material

Crystal structure: contains datablocks I, global. DOI: 10.1107/S1600536809038318/lh2907sup1.cif

Structure factors: contains datablocks I. DOI: 10.1107/S1600536809038318/lh2907Isup2.hkl

Additional supplementary materials:  crystallographic information; 3D view; checkCIF report

## Figures and Tables

**Fig. 1.**
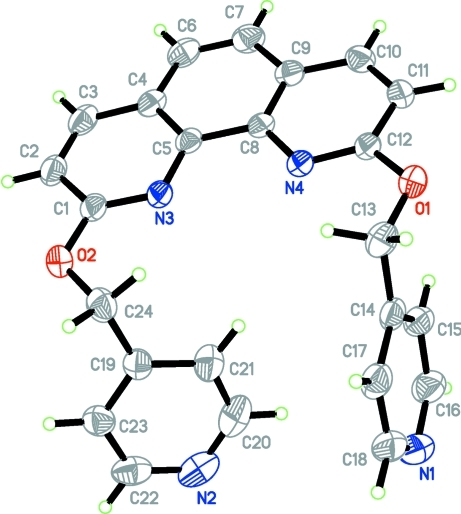
Molecular structure of title compound with the atom-numbering scheme. Displacement ellipsoids are drawn at the 30% probability level.

## supplementary crystallographic information

### Comment

Derivatives of 1,10-phenanthroline play an important role in modern coordination chemistry and a number of the derivatives (e.g. Liu *et al.*, 2008; Zhang *et al.* 2008) have been synthesized and used as ligands to prepare new complexes. Herein we report the crystal structure of the title compound.

The molecular structure is shown in Fig. 1. There are two π–π stacking interactions involving 1,10-phenathroline ring systems, one involving symmetry related pyridine rings with the relevant distances being *Cg*1···*Cg*2^i^ = 3.6101 (11) Å and *Cg*1···*Cg*2^i^_perp_ = 3.373 Å and α = 1.63°; another from the adjacent pyridine rings and the benzene rings with the relevant distances being *Cg*2···*Cg*3^i^ = 3.5864 (11) Å and *Cg*2···*Cg*3^i^_perp_ = 3.383 Å and α = 1.29° [symmetry code: (i) 1 - *x*, -*y*, 1 - *z*; *Cg*1, *Cg*2 and *Cg*3 are the centroids of C1—C5/N3 ring, C8—C12/N4 ring and C4—C9 ring, respectively; *Cg*i···*Cg*j_perp_ is the perpendicular distance from ring *Cg*i to ring *Cg*j^i^; α is the dihedral angle between ring plane *Cg*i and ring plane *Cg*j^i^].

### Experimental

Powdered 2,9-Bis((pyridin-4-yl)methoxy)-1,10-phenanthroline (0.1025 g, 0.260 mmol) was dissolved in 15 ml methanol and yellow single crystals were obtained after the filtrate had been allowed to stand at room temperature for four weeks.

### Refinement

All H atoms were placed in calculated positions and refined as riding with C—H = 0.97 Å for methylene groups and C—H = 0.93 Å for other H atoms and with *U*_iso_(H) = 1.2*U*_eq_(C).

### Crystal data

**Table:**

C_24_H_18_N_4_O_2_	*F*(000) = 824
*M**_r_* = 394.42	*D*_x_ = 1.352 Mg m^−^^3^
Monoclinic, *P*2_1_/*c*	Mo *K*α radiation, λ = 0.71073 Å
Hall symbol: -P 2ybc	Cell parameters from 2776 reflections
*a* = 12.701 (2) Å	θ = 2.5–25.2°
*b* = 16.418 (3) Å	µ = 0.09 mm^−^^1^
*c* = 9.7443 (16) Å	*T* = 298 K
β = 107.535 (2)°	Block, yellow
*V* = 1937.5 (6) Å^3^	0.51 × 0.40 × 0.38 mm
*Z* = 4	

### Data collection

**Table:**

Bruker SMART APEX CCD diffractometer	3530 independent reflections
Radiation source: fine-focus sealed tube	2603 reflections with *I* > 2σ(*I*)
graphite	*R*_int_ = 0.028
φ and ω scans	θ_max_ = 25.3°, θ_min_ = 1.7°
Absorption correction: multi-scan (*SADABS*; Sheldrick, 1996)	*h* = −15→11
*T*_min_ = 0.956, *T*_max_ = 0.967	*k* = −14→19
9860 measured reflections	*l* = −10→11

### Refinement

**Table:**

Refinement on *F*^2^	Primary atom site location: structure-invariant direct methods
Least-squares matrix: full	Secondary atom site location: difference Fourier map
*R*[*F*^2^ > 2σ(*F*^2^)] = 0.043	Hydrogen site location: inferred from neighbouring sites
*wR*(*F*^2^) = 0.113	H-atom parameters constrained
*S* = 1.03	*w* = 1/[σ^2^(*F*_o_^2^) + (0.0569*P*)^2^ + 0.0745*P*] where *P* = (*F*_o_^2^ + 2*F*_c_^2^)/3
3530 reflections	(Δ/σ)_max_ = 0.002
271 parameters	Δρ_max_ = 0.13 e Å^−^^3^
0 restraints	Δρ_min_ = −0.16 e Å^−^^3^

### Special details

**Table:**

Geometry. All e.s.d.'s (except the e.s.d. in the dihedral angle between two l.s. planes) are estimated using the full covariance matrix. The cell e.s.d.'s are taken into account individually in the estimation of e.s.d.'s in distances, angles and torsion angles; correlations between e.s.d.'s in cell parameters are only used when they are defined by crystal symmetry. An approximate (isotropic) treatment of cell e.s.d.'s is used for estimating e.s.d.'s involving l.s. planes.
Refinement. Refinement of *F*^2^ against ALL reflections. The weighted *R*-factor *wR* and goodness of fit *S* are based on *F*^2^, conventional *R*-factors *R* are based on *F*, with *F* set to zero for negative *F*^2^. The threshold expression of *F*^2^ > σ(*F*^2^) is used only for calculating *R*-factors(gt) *etc*. and is not relevant to the choice of reflections for refinement. *R*-factors based on *F*^2^ are statistically about twice as large as those based on *F*, and *R*- factors based on ALL data will be even larger.

### Fractional atomic coordinates and isotropic or equivalent isotropic displacement parameters (Å^2^)

**Table:**

	*x*	*y*	*z*	*U*_iso_*/*U*_eq_	
C1	0.50194 (13)	0.23897 (10)	0.53260 (16)	0.0523 (4)	
C2	0.61170 (14)	0.25413 (11)	0.53679 (18)	0.0622 (5)	
H2	0.6488	0.3003	0.5819	0.075*	
C3	0.66137 (14)	0.19987 (12)	0.47360 (18)	0.0626 (5)	
H3	0.7339	0.2085	0.4743	0.075*	
C4	0.60421 (12)	0.12976 (10)	0.40609 (16)	0.0524 (4)	
C5	0.49508 (11)	0.12005 (9)	0.40882 (14)	0.0449 (4)	
C6	0.65165 (13)	0.07031 (12)	0.33719 (18)	0.0643 (5)	
H6	0.7239	0.0769	0.3353	0.077*	
C7	0.59392 (14)	0.00485 (12)	0.27477 (19)	0.0646 (5)	
H7	0.6268	−0.0333	0.2301	0.078*	
C8	0.43337 (11)	0.04990 (9)	0.34246 (15)	0.0441 (4)	
C9	0.48333 (12)	−0.00739 (10)	0.27554 (16)	0.0516 (4)	
C10	0.42012 (14)	−0.07576 (11)	0.21217 (17)	0.0600 (4)	
H10	0.4501	−0.1146	0.1653	0.072*	
C11	0.31631 (14)	−0.08512 (10)	0.21916 (17)	0.0579 (4)	
H11	0.2737	−0.1300	0.1780	0.069*	
C12	0.27507 (12)	−0.02471 (10)	0.29082 (16)	0.0486 (4)	
C13	0.12405 (13)	0.01882 (12)	0.37343 (18)	0.0623 (5)	
H13A	0.1829	0.0488	0.4413	0.075*	
H13B	0.0830	−0.0100	0.4276	0.075*	
C14	0.04897 (11)	0.07741 (10)	0.27374 (18)	0.0542 (4)	
C15	0.03967 (13)	0.08218 (11)	0.12927 (18)	0.0610 (5)	
H15	0.0832	0.0492	0.0906	0.073*	
C16	−0.03412 (15)	0.13578 (12)	0.0426 (2)	0.0752 (5)	
H16	−0.0389	0.1373	−0.0545	0.090*	
C17	−0.01738 (13)	0.12956 (12)	0.3232 (2)	0.0675 (5)	
H17	−0.0141	0.1296	0.4199	0.081*	
C18	−0.08782 (15)	0.18100 (14)	0.2280 (3)	0.0829 (6)	
H18	−0.1313	0.2155	0.2639	0.100*	
C19	0.26496 (12)	0.31914 (10)	0.46362 (16)	0.0526 (4)	
C20	0.13577 (17)	0.31235 (18)	0.2316 (2)	0.0918 (7)	
H20	0.0968	0.2803	0.1542	0.110*	
C21	0.20466 (15)	0.27345 (13)	0.34918 (19)	0.0721 (5)	
H21	0.2103	0.2170	0.3512	0.087*	
C22	0.17931 (16)	0.43588 (14)	0.3319 (2)	0.0781 (6)	
H22	0.1710	0.4922	0.3281	0.094*	
C23	0.25156 (14)	0.40236 (12)	0.4528 (2)	0.0636 (5)	
H23	0.2914	0.4358	0.5275	0.076*	
C24	0.34284 (14)	0.28164 (11)	0.59467 (17)	0.0622 (5)	
H24A	0.3305	0.3051	0.6799	0.075*	
H24B	0.3288	0.2236	0.5950	0.075*	
N1	−0.09879 (13)	0.18537 (11)	0.0876 (2)	0.0860 (5)	
N2	0.12073 (13)	0.39253 (15)	0.2202 (2)	0.0891 (6)	
N3	0.44458 (9)	0.17568 (8)	0.47191 (12)	0.0472 (3)	
N4	0.32756 (9)	0.04086 (7)	0.34768 (12)	0.0455 (3)	
O1	0.17152 (8)	−0.03913 (7)	0.29945 (12)	0.0615 (3)	
O2	0.45550 (9)	0.29496 (7)	0.59879 (12)	0.0660 (3)	

### Atomic displacement parameters (Å^2^)

**Table:**

	*U*^11^	*U*^22^	*U*^33^	*U*^12^	*U*^13^	*U*^23^
C1	0.0513 (9)	0.0475 (10)	0.0472 (9)	−0.0005 (8)	−0.0017 (7)	0.0046 (8)
C2	0.0538 (10)	0.0537 (11)	0.0650 (11)	−0.0114 (8)	−0.0031 (8)	0.0087 (9)
C3	0.0434 (9)	0.0704 (12)	0.0662 (11)	−0.0102 (9)	0.0049 (8)	0.0173 (10)
C4	0.0418 (8)	0.0599 (11)	0.0521 (9)	−0.0008 (8)	0.0091 (7)	0.0135 (8)
C5	0.0419 (8)	0.0474 (9)	0.0402 (8)	0.0014 (7)	0.0043 (6)	0.0088 (7)
C6	0.0431 (9)	0.0840 (14)	0.0664 (11)	0.0034 (9)	0.0176 (8)	0.0102 (10)
C7	0.0553 (10)	0.0762 (13)	0.0658 (11)	0.0124 (10)	0.0234 (8)	−0.0016 (9)
C8	0.0400 (8)	0.0485 (9)	0.0401 (8)	0.0042 (7)	0.0064 (6)	0.0061 (7)
C9	0.0500 (9)	0.0556 (10)	0.0473 (9)	0.0088 (8)	0.0118 (7)	0.0023 (8)
C10	0.0637 (11)	0.0539 (11)	0.0575 (10)	0.0119 (8)	0.0109 (8)	−0.0084 (8)
C11	0.0591 (10)	0.0460 (10)	0.0590 (10)	0.0009 (8)	0.0032 (8)	−0.0056 (8)
C12	0.0421 (8)	0.0457 (9)	0.0513 (9)	−0.0006 (7)	0.0038 (7)	0.0036 (7)
C13	0.0459 (9)	0.0767 (12)	0.0654 (10)	−0.0077 (9)	0.0185 (8)	−0.0004 (9)
C14	0.0365 (8)	0.0619 (11)	0.0620 (10)	−0.0109 (7)	0.0114 (7)	−0.0110 (8)
C15	0.0481 (9)	0.0693 (12)	0.0631 (11)	0.0026 (8)	0.0130 (8)	−0.0089 (9)
C16	0.0634 (11)	0.0867 (15)	0.0662 (11)	0.0084 (11)	0.0054 (9)	−0.0062 (11)
C17	0.0475 (10)	0.0841 (14)	0.0718 (11)	−0.0079 (9)	0.0192 (9)	−0.0200 (10)
C18	0.0513 (11)	0.0878 (16)	0.1067 (18)	0.0102 (10)	0.0193 (11)	−0.0274 (13)
C19	0.0510 (9)	0.0587 (11)	0.0521 (9)	−0.0031 (8)	0.0216 (7)	−0.0066 (8)
C20	0.0708 (14)	0.122 (2)	0.0703 (13)	−0.0159 (13)	0.0028 (10)	−0.0075 (14)
C21	0.0717 (12)	0.0748 (13)	0.0633 (11)	−0.0110 (10)	0.0107 (9)	−0.0086 (10)
C22	0.0615 (12)	0.0805 (14)	0.1001 (16)	0.0159 (11)	0.0363 (12)	0.0180 (13)
C23	0.0602 (10)	0.0630 (12)	0.0709 (11)	0.0037 (9)	0.0246 (9)	−0.0052 (9)
C24	0.0737 (12)	0.0593 (11)	0.0532 (10)	−0.0022 (9)	0.0184 (8)	−0.0102 (8)
N1	0.0628 (10)	0.0889 (13)	0.0937 (13)	0.0183 (9)	0.0047 (9)	−0.0108 (10)
N2	0.0573 (10)	0.1221 (17)	0.0853 (12)	0.0093 (11)	0.0178 (9)	0.0238 (13)
N3	0.0450 (7)	0.0455 (8)	0.0446 (7)	−0.0008 (6)	0.0038 (5)	0.0012 (6)
N4	0.0408 (7)	0.0447 (8)	0.0469 (7)	0.0008 (6)	0.0070 (5)	0.0016 (6)
O1	0.0450 (6)	0.0579 (7)	0.0769 (8)	−0.0079 (5)	0.0111 (5)	−0.0047 (6)
O2	0.0624 (8)	0.0551 (7)	0.0686 (7)	−0.0029 (6)	0.0017 (6)	−0.0150 (6)

### Geometric parameters (Å, °)

**Table:**

C1—N3	1.3041 (19)	C13—H13A	0.9700
C1—O2	1.3558 (19)	C13—H13B	0.9700
C1—C2	1.405 (2)	C14—C15	1.379 (2)
C2—C3	1.343 (2)	C14—C17	1.386 (2)
C2—H2	0.9300	C15—C16	1.374 (2)
C3—C4	1.413 (2)	C15—H15	0.9300
C3—H3	0.9300	C16—N1	1.322 (2)
C4—C5	1.403 (2)	C16—H16	0.9300
C4—C6	1.418 (2)	C17—C18	1.369 (3)
C5—N3	1.3641 (19)	C17—H17	0.9300
C5—C8	1.433 (2)	C18—N1	1.335 (3)
C6—C7	1.339 (2)	C18—H18	0.9300
C6—H6	0.9300	C19—C21	1.370 (2)
C7—C9	1.421 (2)	C19—C23	1.377 (2)
C7—H7	0.9300	C19—C24	1.492 (2)
C8—N4	1.3681 (18)	C20—N2	1.330 (3)
C8—C9	1.401 (2)	C20—C21	1.372 (3)
C9—C10	1.410 (2)	C20—H20	0.9300
C10—C11	1.349 (2)	C21—H21	0.9300
C10—H10	0.9300	C22—N2	1.325 (3)
C11—C12	1.402 (2)	C22—C23	1.372 (3)
C11—H11	0.9300	C22—H22	0.9300
C12—N4	1.2992 (18)	C23—H23	0.9300
C12—O1	1.3636 (17)	C24—O2	1.436 (2)
C13—O1	1.432 (2)	C24—H24A	0.9700
C13—C14	1.490 (2)	C24—H24B	0.9700
			
N3—C1—O2	119.50 (14)	C15—C14—C17	116.50 (16)
N3—C1—C2	124.64 (16)	C15—C14—C13	122.91 (15)
O2—C1—C2	115.86 (15)	C17—C14—C13	120.58 (16)
C3—C2—C1	117.91 (16)	C16—C15—C14	119.77 (17)
C3—C2—H2	121.0	C16—C15—H15	120.1
C1—C2—H2	121.0	C14—C15—H15	120.1
C2—C3—C4	120.48 (15)	N1—C16—C15	124.57 (19)
C2—C3—H3	119.8	N1—C16—H16	117.7
C4—C3—H3	119.8	C15—C16—H16	117.7
C5—C4—C3	117.10 (15)	C18—C17—C14	119.14 (17)
C5—C4—C6	119.59 (15)	C18—C17—H17	120.4
C3—C4—C6	123.31 (15)	C14—C17—H17	120.4
N3—C5—C4	122.25 (14)	N1—C18—C17	124.95 (18)
N3—C5—C8	118.29 (12)	N1—C18—H18	117.5
C4—C5—C8	119.46 (14)	C17—C18—H18	117.5
C7—C6—C4	120.99 (15)	C21—C19—C23	117.07 (17)
C7—C6—H6	119.5	C21—C19—C24	122.29 (16)
C4—C6—H6	119.5	C23—C19—C24	120.63 (15)
C6—C7—C9	121.18 (16)	N2—C20—C21	124.7 (2)
C6—C7—H7	119.4	N2—C20—H20	117.6
C9—C7—H7	119.4	C21—C20—H20	117.6
N4—C8—C9	122.16 (14)	C19—C21—C20	119.0 (2)
N4—C8—C5	118.70 (13)	C19—C21—H21	120.5
C9—C8—C5	119.14 (13)	C20—C21—H21	120.5
C8—C9—C10	117.42 (14)	N2—C22—C23	123.7 (2)
C8—C9—C7	119.64 (15)	N2—C22—H22	118.2
C10—C9—C7	122.93 (15)	C23—C22—H22	118.2
C11—C10—C9	120.31 (15)	C22—C23—C19	119.93 (18)
C11—C10—H10	119.8	C22—C23—H23	120.0
C9—C10—H10	119.8	C19—C23—H23	120.0
C10—C11—C12	117.52 (15)	O2—C24—C19	111.04 (13)
C10—C11—H11	121.2	O2—C24—H24A	109.4
C12—C11—H11	121.2	C19—C24—H24A	109.4
N4—C12—O1	119.88 (14)	O2—C24—H24B	109.4
N4—C12—C11	125.30 (14)	C19—C24—H24B	109.4
O1—C12—C11	114.82 (14)	H24A—C24—H24B	108.0
O1—C13—C14	112.62 (14)	C16—N1—C18	115.06 (17)
O1—C13—H13A	109.1	C22—N2—C20	115.61 (18)
C14—C13—H13A	109.1	C1—N3—C5	117.62 (13)
O1—C13—H13B	109.1	C12—N4—C8	117.24 (13)
C14—C13—H13B	109.1	C12—O1—C13	118.41 (12)
H13A—C13—H13B	107.8	C1—O2—C24	117.13 (12)
			
N3—C1—C2—C3	0.2 (2)	C14—C15—C16—N1	0.5 (3)
O2—C1—C2—C3	179.44 (14)	C15—C14—C17—C18	0.8 (2)
C1—C2—C3—C4	−0.3 (2)	C13—C14—C17—C18	−178.10 (16)
C2—C3—C4—C5	−0.1 (2)	C14—C17—C18—N1	0.2 (3)
C2—C3—C4—C6	179.82 (15)	C23—C19—C21—C20	0.4 (2)
C3—C4—C5—N3	0.7 (2)	C24—C19—C21—C20	−179.17 (16)
C6—C4—C5—N3	−179.19 (13)	N2—C20—C21—C19	−1.4 (3)
C3—C4—C5—C8	−179.77 (13)	N2—C22—C23—C19	−1.0 (3)
C6—C4—C5—C8	0.3 (2)	C21—C19—C23—C22	0.7 (2)
C5—C4—C6—C7	−0.1 (2)	C24—C19—C23—C22	−179.74 (15)
C3—C4—C6—C7	179.96 (15)	C21—C19—C24—O2	106.35 (17)
C4—C6—C7—C9	−0.1 (3)	C23—C19—C24—O2	−73.16 (19)
N3—C5—C8—N4	−1.30 (19)	C15—C16—N1—C18	0.5 (3)
C4—C5—C8—N4	179.19 (12)	C17—C18—N1—C16	−0.8 (3)
N3—C5—C8—C9	179.21 (12)	C23—C22—N2—C20	0.1 (3)
C4—C5—C8—C9	−0.3 (2)	C21—C20—N2—C22	1.1 (3)
N4—C8—C9—C10	0.2 (2)	O2—C1—N3—C5	−178.81 (12)
C5—C8—C9—C10	179.67 (13)	C2—C1—N3—C5	0.4 (2)
N4—C8—C9—C7	−179.36 (13)	C4—C5—N3—C1	−0.9 (2)
C5—C8—C9—C7	0.1 (2)	C8—C5—N3—C1	179.62 (12)
C6—C7—C9—C8	0.1 (2)	O1—C12—N4—C8	176.56 (12)
C6—C7—C9—C10	−179.45 (16)	C11—C12—N4—C8	−2.9 (2)
C8—C9—C10—C11	−1.1 (2)	C9—C8—N4—C12	1.7 (2)
C7—C9—C10—C11	178.44 (15)	C5—C8—N4—C12	−177.73 (12)
C9—C10—C11—C12	0.1 (2)	N4—C12—O1—C13	−0.8 (2)
C10—C11—C12—N4	2.1 (2)	C11—C12—O1—C13	178.80 (13)
C10—C11—C12—O1	−177.47 (13)	C14—C13—O1—C12	97.39 (15)
O1—C13—C14—C15	−8.9 (2)	N3—C1—O2—C24	−1.5 (2)
O1—C13—C14—C17	169.93 (14)	C2—C1—O2—C24	179.22 (13)
C17—C14—C15—C16	−1.1 (2)	C19—C24—O2—C1	−88.63 (16)
C13—C14—C15—C16	177.76 (15)		
